# Lysophosphatidic Acid Is Associated with Atherosclerotic Plaque Instability by Regulating NF-κB Dependent Matrix Metalloproteinase-9 Expression via LPA_2_ in Macrophages

**DOI:** 10.3389/fphys.2017.00266

**Published:** 2017-04-27

**Authors:** Chun Gu, Fang Wang, Zhenwen Zhao, Hongyue Wang, Xiangfeng Cong, Xi Chen

**Affiliations:** ^1^State Key Laboratory of Cardiovascular Disease, Fuwai Hospital, National Center for Cardiovascular Diseases, Chinese Academy of Medical Sciences and Peking Union Medical CollegeBeijing, China; ^2^Key Laboratory of Analytical Chemistry for Living Biosystems, Institute of Chemistry Chinese Academy of ScienceBeijing, China; ^3^Department of Pathology, Fuwai Hospital, National Center for Cardiovascular Diseases, Chinese Academy of Medical Sciences and Peking Union Medical CollegeBeijing, China

**Keywords:** lysophosphatidic acid, matrix metalloproteinase-9, macrophages, LPA_2_, coronary atherosclerotic plaques

## Abstract

Lysophosphatidic acid (LPA), one of the simplest phospholipid signaling molecules, participates in formation and disruption of atherosclerotic plaque. Matrix metalloproteinases (MMPs) contribute to atherosclerotic plaque rupture by involving in extracellular matrix (ECM) degradation and then thinning fibrous cap. Our previous study demonstrated that macrophage-derived MMP-9 was associated with coronary plaque instability, but the relationship between LPA and MMP-9 remains unclear. The present work therefore aimed at elucidating association between LPA and MMP-9 and the regulation mechanism of LPA on MMP-9 in macrophages. We found that plasma LPA and MMP-9 levels were correlated positively (*r* = 0.31, *P* < 0.05) and both elevated significantly in patients with acute myocardial infarct (AMI). Consistent with peripheral blood levels, histochemical staining indicated that autotaxin (ATX), LPA-producing ectoenzyme, and MMP-9 were expressed frequently in the necrotic core and fibrous cap of human unstable plaques, which might increase the instability of plaque. Experiments *in vitro* were done with THP-1-derived macrophages and showed that LPA enhanced the expression, secretion and activity of MMP-9 in a time- and dose-dependent manner. Induction of LPA on pro-MMP-9 and active-MMP-9 was confirmed in human peripheral blood monocyte-derived macrophages. PDTC, NF-κB inhibitor, but not inhibitor of AP-1 and PPARγ, effectively prevented LPA-induced MMP-9 expression and NF-κB p65 siRNA decreased MMP-9 transcription, confirming that LPA might induce MMP-9 elevation by activating NF-κB pathway. In addition, knockdown of LPA_2_ attenuated LPA-induced MMP-9 expression and nucleus p65 levels. These findings revealed that LPA upregulated the expression of MMP-9 through activating NF-κB pathway in the LPA_2_ dependent manner, hence blocking LPA receptors signaling may provide therapeutic strategy to target plaque destabilization.

## Introduction

Acute coronary syndromes (ACS) is a sudden attack without warning, even life-threatening as a result of the atherosclerosis, one of the leading causes of cardiovascular death worldwide. A postmortem study shows that 75% of ACS arises from atherosclerosis plaque rupture (Narula et al., 2013), but the complicated mechanisms underlying plaque destabilization remain fragmentary. Recent evidence suggests that stable atherosclerotic plaques are rich in smooth muscle cells (SMCs) and collagen, whereas unstable plaques are characterized by a large necrotic core covered with a thin fibrous cap and a highly inflammatory cell content (Libby et al., 2011; Silvestre-Roig et al., 2014). Abundant macrophage infiltration and extracellular matrix (ECM) degradation are mainly contributed to necrotic core expansion and fibrous cap thinning, respectively (Moore and Tabas, 2011).

Matrix metalloproteinases (MMPs) are zinc-dependent enzymes and involved in ECM degradation and remodeling, which may hasten plaque disruption (Ketelhuth and Back, 2011; Muller et al., 2014). MMPs are produced by many vascular wall cells, but macrophages are critical in human plaques (Newby, 2008). It has been reported that elevated levels of MMP-1, -3, -8, -9, -12, and -13 are found in macrophage-rich regions of human atherosclerotic plaques (Sukhova et al., 1999; Morgan et al., 2004; Higashikata et al., 2006; Sluijter et al., 2006; Quillard et al., 2011). In particular, the increased content and activity of MMP-9 are associated with plaque stability. ApoE-deficient mice model indicates that the loss of MMP-9 reduces impaired macrophage infiltration and collagen deposition (Luttun et al., 2004), whereas the overexpression of MMP-9 promotes intraplaque hemorrhage and induces acute plaque disruption (de Nooijer et al., 2006; Gough et al., 2006). Our previous study demonstrated macrophage-derived MMP-9 was associated with coronary plaque instability (Fan et al., 2014), and therefore it is of great interest to discuss the regulation mechanism of MMP-9.

Lysophosphatidic acid (LPA), generated by autotaxin (ATX) (Moolenaar and Perrakis, 2011), is a bioactive and endogenous phospholipid signaling factor, regulating cell proliferation, differentiation, migration, survival and apoptosis through the activation of at least six known G protein-coupled receptors (GPCRs), LPA_1_–LPA_6_ (Yung et al., 2014). It is found that LPA progressively accumulates in human and mouse atherosclerotic plaques in contrast with normal vessels (Bot et al., 2010; Dohi et al., 2013). Actually, LPA participates in the most processes of atherogenesis and thrombosis, such as promoting adhesion molecule expression in endothelial cells (ECs) (Lee et al., 2004), stimulating SMCs migration (Damirin et al., 2007), inducing foam cell formation (Chang et al., 2008). However, whether LPA plays a role in ECM degradation and what's the downstream signaling pathway in macrophages have not been fully elucidated.

In the present study, we observed the correlation between circulating LPA and MMP-9 levels as well as expression in the advanced atherosclerotic plaques. Furthermore, we also investigated the detailed mechanisms of LPA on MMP-9 in macrophages—via NF-κB signaling in the LPA_2_ dependent manner, and thus LPA receptors (LPARs) might be potential therapeutic target to prevent plaque rupture.

## Materials and methods

### Study population

Twenty-four patients with unstable angina (UA) and twenty-four patients with non-ST-segment elevation myocardial infarction (NSTEMI) were recruited in Fuwai Hospital (Beijing, China) between March 2013 and January 2014 (Table 1). The diagnoses of UA/NSTEMI were all in line with the standards of the Chinese Medical Society of Cardiology. Twenty-four healthy persons served as a control group with normal echocardiograms (ECGs), negative exercise stress ECGs based on physical examination and laboratory tests and without coronary heart disease history. Patients with inflammatory diseases, acute myocardial injury, severe heart failure, malignant disease, impaired liver function, renal failure, or recent surgery were excluded.

**Table 1:** **Characteristics of patients and controls**.

	**Controls (*n* = 24)**	**UA (*n* = 24)**	**NSTEMI (*n* = 24)**	***p***
Age (years)	60.2 ± 8.7	61.4 ± 9.3	61.5 ± 8.8	ns
Men (%)	16 (64.0%)	18 (75.0%)	16 (66.7%)	ns
Body mass index (kg/m^2^)	25.4 ± 3.2	25.4 ± 3.3	25.2 ± 3.4	ns
Smoking	9 (36.0%)	14 (58.3%)	15 (62.5%)	ns
Hypertension (%)	13 (52.0%)	21 (87.5%)	19 (79.2%)	0.014
Diabetes Mellitus (%)	5 (20.0%)	8 (33.3%)	10 (41.7%)	ns
Family history of coronary artery disease (%)	6 (24.0%)	10 (41.7%)	9 (37.5%)	ns

*Data are presented as mean ± SD or number (%)*.

*UA, unstable angina; NSTEMI, non-ST-segment elevation myocardial infarction*.

The coronary atherosclerotic plaques were collected from explanted hearts donated by three patients undergoing heart transplantation and stored in liquid nitrogen in Fuwai Hospital. According to histomorphologic features, atherosclerotic plaques were classified as follows: unstable plaques were defined as the lesions with thin fibrous cap (<65 μm) covering large lipid core (>40% plaque volume); stable plaques were described as the lesions with well-matured necrotic core (<40% plaque volume) covered by thick fibrous cap (>65 μm). All specimens were carefully perfusion-fixed in 10% buffered formalin.

The study protocol conformed to the Declaration of Helsinki and was approved by the ethics review board of Fu Wai Hospital and all the patients gave written informed consent.

### Hematoxylin-eosin (H&E) and immunohistochemistry (IHC) staining

Formalin-fixed paraffin embedded tissue were cuted into 4-μm-thick sections and immersed in xylene and rehydrated with graded alcohols and subjected to H&E staining before examination by light microscopy.

IHC was performed by the standard protocol. Briefly, antigen retrieval was performed in 0.01 M citrate buffer (pH 6.0), followed by quenching of endogenous peroxidase activity with 3% H_2_O_2_ in methanol. Sections were then incubated with MMP-9 (Abcam, Cambridge, UK) and ATX (as described Li and Zhang, 2009) primary antibodies overnight at 4°C. Powervision horseradish peroxidase-IgG was used as secondary antibody. Sections were visualized using diaminobenzidine and counter stained with hematoxylin.

### Cell culture

Human monocytic cell line THP-1 (a gift from Prof. Junjie Zhang, Beijing Normal University, China) and maintained at a density of 10^6^/ml in RPMI 1640 medium (Gibco, CA, USA) containing 10% fetal bovine serum (FBS) and 1% penicillin/Streptomycin, and then incubated at 37°C with 5% CO^2^. Cells were cultured in six-well plates for 48 h in the presence of phorbol 12-myristate 13-acetate (PMA) (100 ng/mL, Sigma, St Louis, USA), which induced the differentiation into adherent macrophages (Aldo et al., 2013). After serum-starvation for 18–24 h, cells were treated with 0 to 50 μM LPA (oleoyl C 18:1, Avanti Polar Lipids, Alabaster, AL), or pretreated 0.1 μM Actinomycin D (Act.D), 0.1 μM Cycloheximide (CHX), 10 μM GW9662 (PPARγ inhibitor), 10 μM PDTC (NF-κB inhibitor) and 10 μM Tan II A (AP-1 inhibitor) for 1 h.

Isolation of peripheral blood mononuclear cells (PBMCs) and culture of macrophages were performed using classical methods (van Grevenynghe et al., 2003). Briefly, PBMCs were isolated through Ficoll-Paque™ PLUS (GE Healthcare, Piscataway, NJ, USA) gradient centrifugation within 1 h after blood collection. Cells were seeded in culture flasks with RPMI 1640 medium supplemented with 10% FBS and 1% penicillin/Streptomycin for 2 h to adhesion. The adherent monocytes were then added recombinant human granulocyte macrophage colony-stimulating factor (rhGM-CSF) (PeproTech, New Jersey, USA) for 7 days.

### Transfection of siRNA for LPA receptors

Small interfering RNAs (siRNAs) for LPA_1_, LPA_2_, and LPA_6_ were transfected using Lipofectamine™ RNAiMAX according to manufacturer's protocols. The target sequences of these siRNAs were listed as follow:

LPA_1_ Stealth siRNA duplexes (LPA_1_-siRNA) targeting sequences: 5′-GAA AUG AGC GCC ACC UUU A-3′and 5′-UAA AGG UGG CGC UCA UUU C -3′; LPA_2_ Stealth siRNA duplexes (LPA_2_-siRNA) targeting sequences: 5′-GGU CAA UGC UGC UGU GUA C-3′ and 5′-GUA CAC AGC AGC AUU GAC C-3′; LPA_6_ Stealth siRNA duplexes (LPA_6_-siRNA) targeting sequences: 5′-UCA GCA UGG UGU UUG UGC UUG GGU U-3′ and 5′-AAC CCA AGC ACA AAC ACC AUG CUG A-3′. NF-κB p65-siRNA targeting sequences: 5′-GCC CUA UCC CUU UAC GUC A-3′ and 5′-UGA CGU AAA GGG AUA GGG C-3′. The scrambled siRNA controls were used as a negative control.

### Quantitative real time PCR (qRT-PCR)

Total RNA was isolated from THP-1 derived macrophages using Trizol according to the manufacture's instruction and then quantified by NANODROP 2000 spectrophotometer. cDNA was generated from 2 μg total RNA using M-MLV reverse transcriptase and oligo (dT) 15 primer. qRT -PCR was performed using SYBR GREEN PCR Master Mix in the Applied Biosystems 7300 (Foster city, CS, USA). All gene specific extron primers used in the present study were as follows:

LPA_1_: 5′-GCT GCC ATC TCT ACT TCC AT-3′ and 5′-CCA TTC TGT GGC AAG ATG CT-3′; LPA_2_: 5′-CAG CGC ATG GCA GAG CAT GT-3′ and 5′-CCA GGA CAT TGC AGG ACT CAC A-3′; LPA_3_: 5′-AAC GTG AGC GGA TGT TCA CT-3′ and 5′-CCG CGA TGA CCA GAG AAT TA-3′; LPA_4_: 5′-TTC GAA CTA ATG TGG AGG AA-3′ and 5′-TGG AAT TGG AAG TCA ATG AA-3′; LPA_5_: 5′-CAG AGC AAC ACG GAG CAC AG-3′ and 5′-CAC CAG AAT CAT GGC ATG GC-3′; LPA_6_: 5′-CTT CAC AAC ACG GAA TTG GC-3′ and 5′-AGT TAA CCA CAC GCC AGT GC-3′; MMP-9: 5′-TGG AAA ATT ATT GCG CCT CT-3′ and 5′-CCT CGA TGA GAC CAT CAA CA-3′; GAPDH: 5′-ACT AGG CGC TCA CTG TTC TC-3′ and 5′-GCC CAA TAC GAC CAA ATC CG-3′; NF-κB p65: 5'-GCG AGA GGA GCA CAG ATA CC-3' and 5'-CTG ATA GCC TGC TCC AGG TC-3'. The relative expression of each gene was normalized to GAPDH by subtracting the corresponding GAPDH threshold cycle (Ct) values using comparative 2^−ΔΔCt^ method.

### Western blot analysis

The treated cells were washed with ice-cold phosphate-buffered saline (PBS) twice and then lysed in lysis buffer (50 mM Tris-HCl, pH 7.5, 150 mM NaCl, 1% Triton X-100, 1 mM EDTA, 1 mM PMSF and 1% sodium deoxycholate) for 30 min on ice. The BCA assay was used to quantify the extract protein concentration. Equal amounts of protein (20 μg/lane) were separated on 10% SDS-PAGE gels by electrophoresis and then transferred to nitrocellulose membranes using semi-dry electroblotting apparatus. The membranes were blocked for 2 h at room temperature in 5% skim milk and subsequently incubated with primary antibodies overnight at 4°C. In the following day, the members were washed and incubated with secondary antibody for 2 h. Protein bands were visualized by FluorChem M system (Protein Simple, USA) and quantified by densitometry with Quantity One software. The target signals were normalized to the GAPDH levels.

### Luciferase reporter assay

THP-1 derived macrophages were cultured on 96-well plates (10^5^ cells/well) for 48 h and stimulated with 10 μM LPA for 24 h. Reporter plasmid REPO™NF-κB and REPO™AP-1 (Genomeditech, Shanghai, China) were transfected using Lipofectamine 2000. Luciferase activity, quantified as relative luminescence units (firefly luminescence/Renilla luminescence), was measured with the Dual-Glo® Luciferase Assay System (Promega, Madison, WI) according to the manufacturer's instructions using a microplate reader (Infinite 200, Tecan).

### Gelatin zymography

The MMPs activities were assayed as described (Zhang et al., 2014). Briefly, 2 × 10^6^ cells in a 6-well plate were cultured in serum-free medium and 10 μL supernatants were loaded on 8% SDS-PAGE gels containing 2 mg/ml gelatin under non-denaturing conditions. The gels were washed with 2.5% Triton X-100 and incubated overnight in buffer (150 mM NaCl, 5 mM CaCl_2_, 50 mM Tris-HCl, pH 7.6) at 37°C. Following staining with Coomassie brilliant blue R250, the gelatinolytic activities were detected as clear bands against a blue background. The scan of the gel was analyzed by densitometry using FluorChem M system (Protein Simple, USA).

### Enzyme linked immunosorbent assay (Elisa)

Levels of MMP-9 in plasma or conditioned medium from THP-1 derived macrophages were quantified by the human MMP-9 Quantikine ELISA Kit (R&D Systems, Minneapolis, MN, USA) according to the manufacturer's instructions. All plasma samples were diluted 1:100 and the conditioned mediums were diluted 1:10 when testing.

### Electrospray ionization tandem mass spectrometry

Detection of plasma LPA levels were carried out by Prof. Zhenwen Zhao (Institute of Chemistry Chinese Academy of Science, Beijing, China) through high performance-liquid-chromatography electrospray ionization tandem mass spectrometry (HPLCESI-MS/MS) as described (Zhao and Xu, 2009).

### Statistical analysis

All statistical data were presented as mean ± SD and categorical variables were represented as percentages. Comparisons among the groups were performed using the chi-square test or one-way ANOVA. Bivariate correlation analysis was used to analyze the relations of plasma LPA and MMP-9 levels. Two-sided *P*-values were used and *P* < 0.05 were considered statistically significant. All the statistical analyses were performed using SPSS version 17.0 (SPSS, Inc., Chicago, IL, USA).

## Results

### The plasma LPA and MMP-9 levels are elevated significantly in patients with acute myocardial infarction

**Figure 1:**
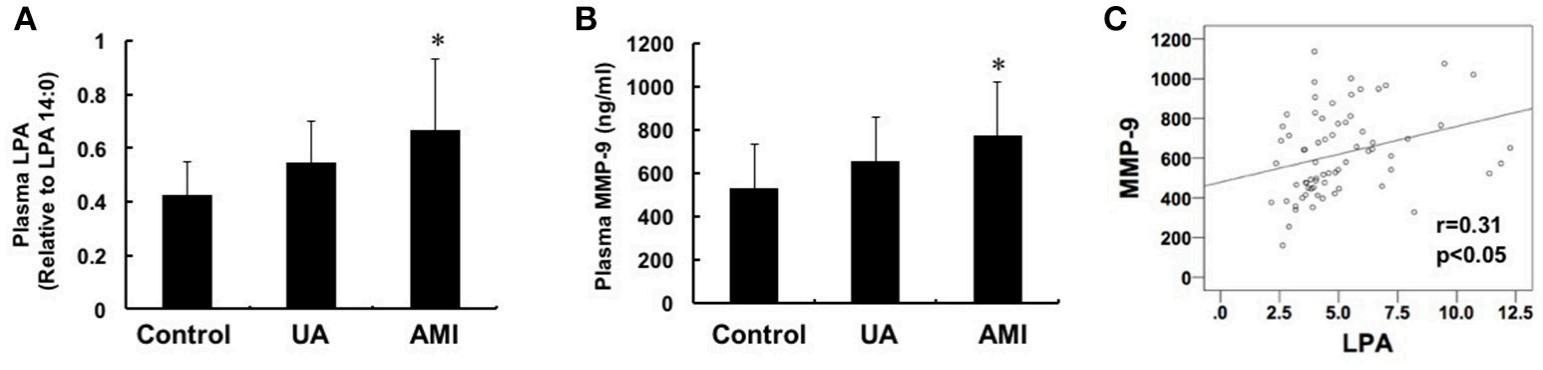
**Changes of plasma LPA and MMP-9 levels among control, unstable angina and acute myocardial infarction groups**. Plasma LPA **(A)** and MMP-9 **(B)** levels were detected by mass spectrometry and ELISA, respectively. The levels of MMP-9 and LPA in the group with AMI were both higher than the control and UA groups (*P* < 0.05). **(C)** Pearson correlation analysis showed that there is a positive correlation between LPA and MMP-9 (*r* = 0.31, *P* < 0.05). ^*^P < 0.05, compared with control.

We measured the plasma levels of LPA and MMP-9 in patients with AMI (*n* = 24), UA (*n* = 24) and asymptomatic controls (*n* = 24) and the data showed that both LPA and MMP-9 with higher levels in plasma in AMI group compared with that in the control group (LPA: 0.66 ± 0.23 vs. 0.42 ± 0.16, *P* < 0.05; MMP-9: 775.5 ± 242.4 vs. 532.1 ± 204.1, *P* < 0.05), but no significant difference was found between patients with UA and controls (Figures 1A,B). Pearson correlation analysis showed that there was a positive correlation between plasma LPA and MMP-9 levels (*r* = 0.31, *P* < 0.05) (Figure 1C).

### The expression of ATX and MMP-9 increases in human unstable plaques

**Figure 2:**
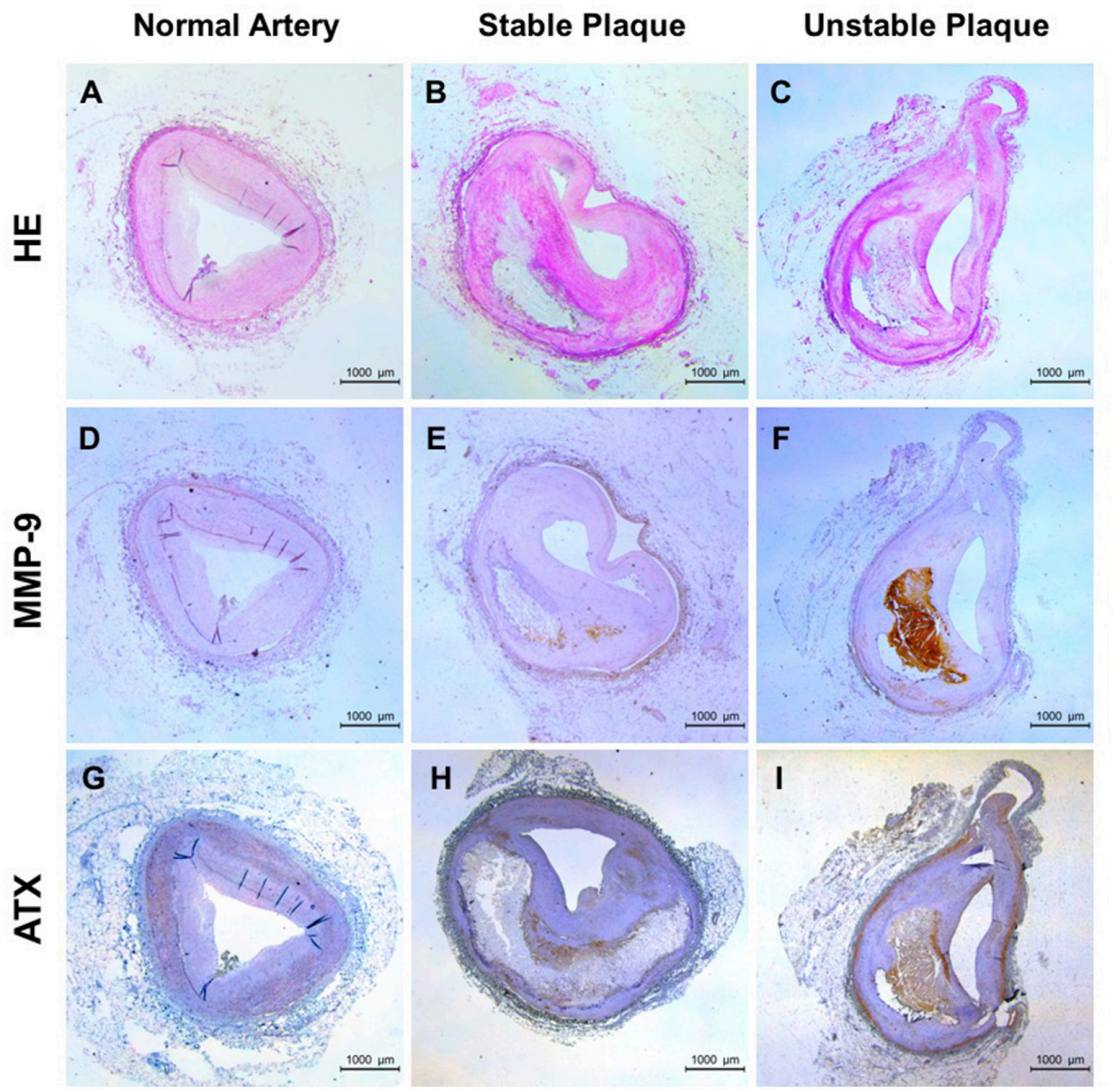
**Expression of ATX and MMP-9 in human coronary plaque tissues**. **(A**,**D**,**G)** for normal arteries; **(B,E,H)** for stable plaques; **(C,F,I)** for unstable plaques; **(A–C)** HE staining; **(D–F)** Immunohistochemical staining of MMP-9; **(G–I)** Immunohistochemical staining of ATX.

The positive correlation between LPA and MMP-9 in the peripheral blood was found, but the presence and localization of them in the local tissue are unclear. ATX, LPA-producing ectoenzyme, were detected because LPA is a bioactive phospholipid molecules and it is difficult to detect in the tissue. As shown in Figures 2A–C, normal artery was characterized by uniform intima-media thickness (IMT) and smooth vascular lumen, stable plaque with large lipid core but thick fibrous cap and the large lipid core and rupture-prone fibrous cap in unstable plaques. The positive staining for MMP-9 was lacked in normal artery (Figure 2D) and mainly located in the shoulder of necrotic core and cap region in stable plaque (Figure 2E). In contrast, unstable plaque showed strong staining for MMP-9 in and surrounding the lesion (Figure 2F). The positive staining for ATX was found in normal artery, stable and unstable plaque and it seemed somewhat a change in distribution. ATX is mainly distributed in the vascular smooth muscle layer and accumulated in the necrotic core and fibrous cap with the increase of plaque instability (Figures 2G–I). Furthermore, distribution patterns of MMP-9 and ATX overlapped in unstable plaque although were different.

### LPA enhances the expression, secretion and activation of MMP-9 in macrophage

**Figure 3:**
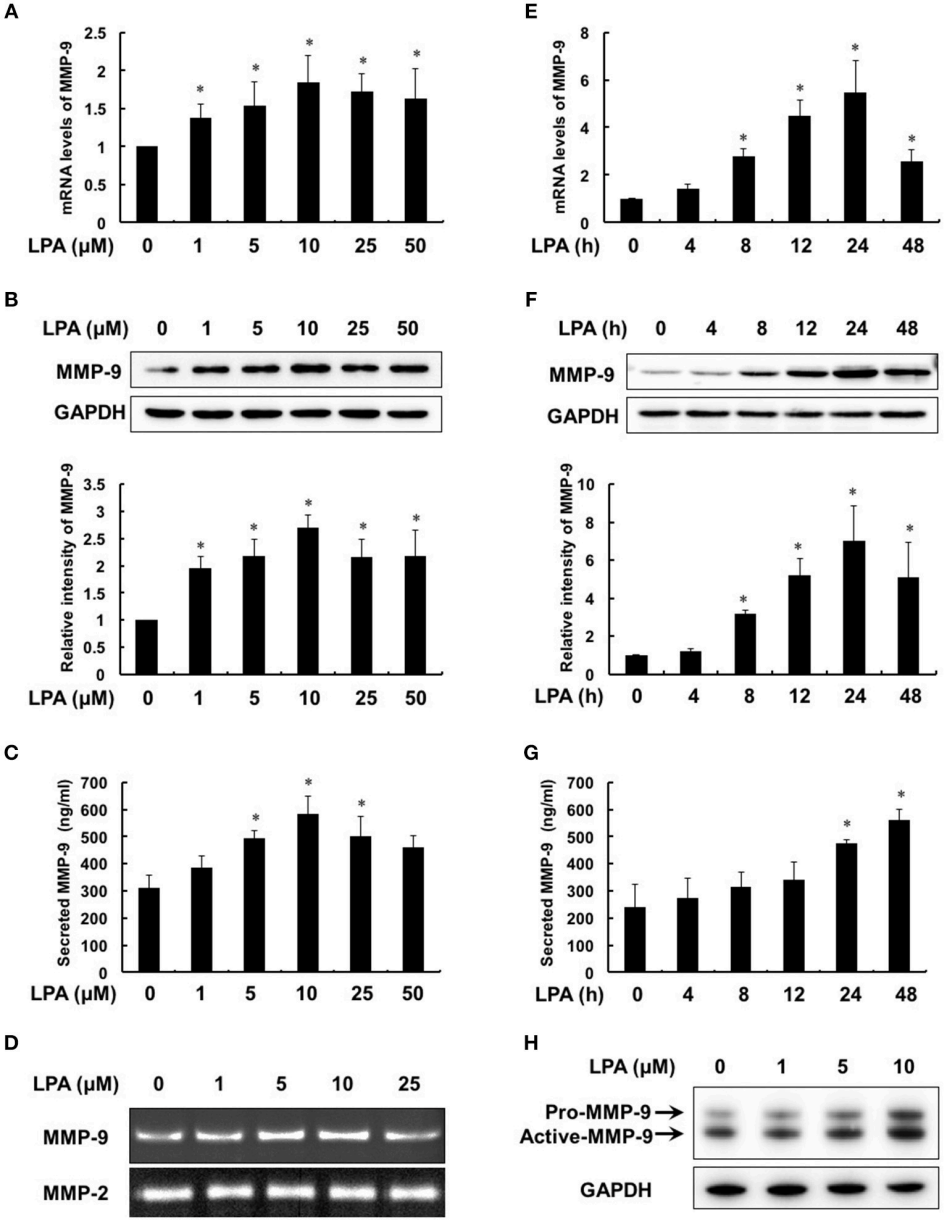
**LPA induced the expression, secretion and activity of MMP-9 in a dose- and time- dependent manner**. Macrophages were treated with the pointed concentrations of LPA for 24 h or with 10 μM LPA for the pointed time course. The mRNA levels **(A**,**E)**, protein levels **(B**,**F)**, secretion **(C**,**G)** and activity **(D)** of MMP-9 were detected by RT-PCR, Western Blot, ELISA and gelatin zymography, respectively. LPA induced MMP-9 expression in human peripheral blood monocyte-derived macrophages **(H)**. Data were expressed as mean ± SD of three independent experiments. ^*^P < 0.05, compared with control.

To evaluate the effect of LPA on MMP-9, we first treated THP-1-derived macrophages with different concentrations of LPA (0, 1, 5, 10, 25, 50 μM) for 24 h. The expression and secretion of MMP-9 increased from 1 μM of LPA in dose-dependent manner and reached maximum at 10 μM of LPA (Figures 3A–C). Meanwhile, Figure 3D revealed that the ability of MMP-9 to degrade collagen was enhanced with the strongest at 10 μM of LPA, while MMP-2 made no responses to LPA. As shown in Figures 3E–G, mRNA levels, protein syntheses and secretion of MMP-9 increased time-dependently with 10 μM LPA for 4–48 h. Additionally, the protein levels of pro-MMP-9 and active-MMP-9 were both increased with LPA treatment in human peripheral blood monocyte-derived macrophages (Figure 3H).

### NF-κB activation is required for LPA-induced MMP-9 expression

**Figure 4:**
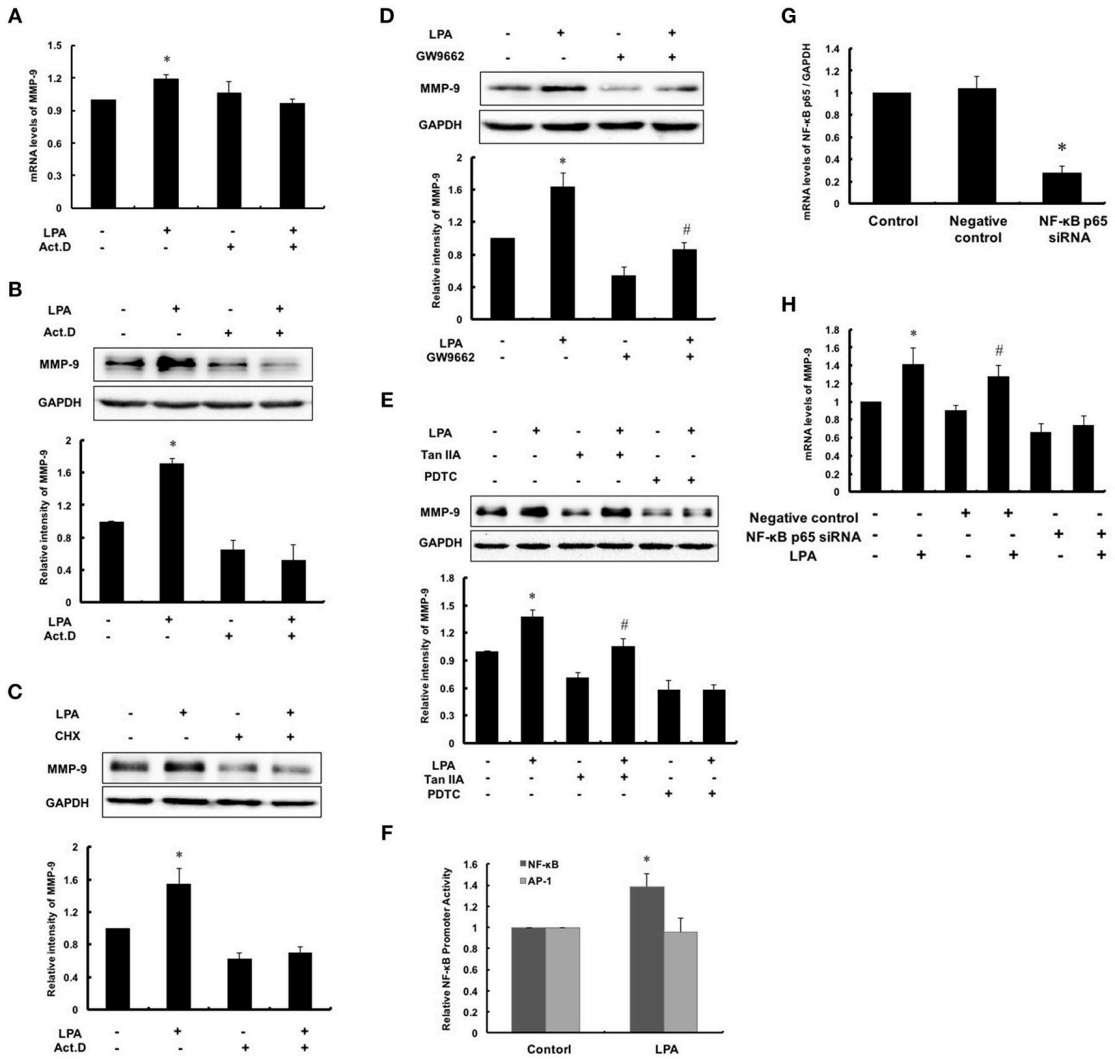
**NF-κB activation was required for LPA-induced MMP-9 expression**. After pretreating with 0.1 μM Act.D **(A,B)** and 0.1 μM CHX **(C)** for 1 h to inhibit mRNA and protein synthesis, and then cells were incubated with 10 μM LPA for 24 h to detect MMP-9 expression. **(D,E)** Cells were pretreated with 10 μM GW9662 (PPARγ inhibitor), 10 μM PDTC (NF-κB inhibitor) and 10 μM TanIIA (AP-1 inhibitor) respectively, and then incubated with 10 μM LPA for 24 h. The expression of MMP-9 was determined. **(F)** The NF-κB and AP-1 promoter activity was measured. The mRNA expression of NF-κB p65 **(G)** and MMP-9 **(H)** were determined after interference of NF-κB p65-siRNA. Data were expressed as mean ± SD of three independent experiments. ^*^P < 0.05, compared with control; ^#^*P* < 0.05, compared with inhibitor treatment groups.

MMPs are regulated at the levels of transcription, secretion, activation and physiological suppression of tissue inhibitor of matrix metalloproteinases (TIMPs). To determine the regulation at transcriptional or post transcriptional, Act.D and CHX were used to inhibit synthesis of nascent mRNA and protein of MMP-9, respectively. As shown in Figures 4A–C, the expression of MMP-9 failed to increase after pretreatment with Act.D or CHX followed by LPA exposure. It indicated that up-regulation of LPA-induced MMP-9 occurred in the de novo synthesis of mRNA, namely transcription. Thus, we analyzed promoter region of MMP-9 and filtered out three possible transcription factors: PPARγ, NF-κB and AP-1. As shown in Figures 4D,E, after pretreatment with PPARγ inhibitor GW9662 and AP-1 inhibitor, the basic levels of MMP-9 reduced, but LPA-induced MMP-9 still increased, indicating that PPARγ and AP-1 were not involved in the regulating process of LPA on MMP-9. In contrast, LPA-induced MMP-9 expression was markedly attenuated by NF-κB inhibitor PDTC and siRNA for NF-κB p65 (Figures 4E,G,H), and transcriptional activity of NF-κB was up-regulated by LPA as expected (Figure 4F).

### LPA induces MMP-9 expression through LPA_2_

**Figure 5:**
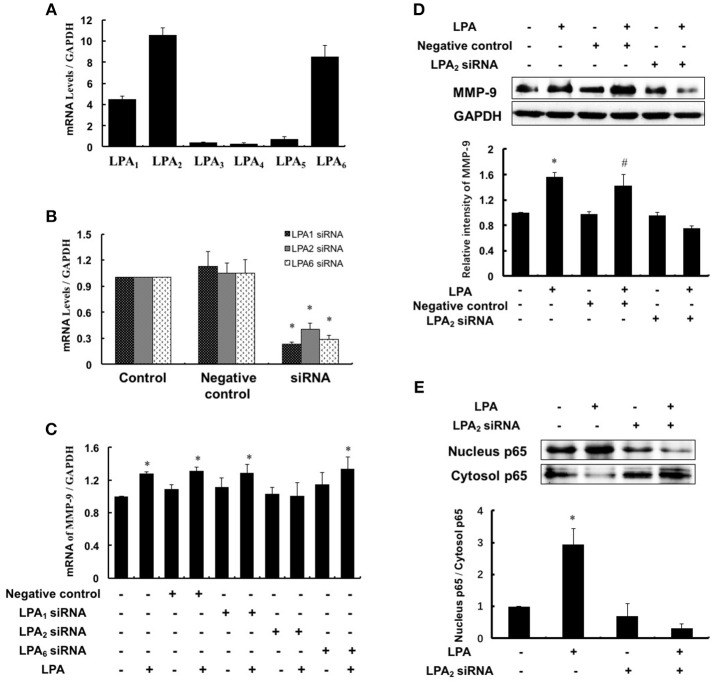
**LPA_2_ mediated LPA-induced MMP-9 expression. (A)** The expression of LPAR was detected in THP-1-derived macrophages. The mRNA expression of LPAR **(B)** and MMP-9 **(C)** were determined after interference of LPA_1_-siRNA, LPA_2_-siRNA and LPA_6_-siRNA, respectively. The expression of MMP-9 **(D)**, nucleus and cytoplasm p65 **(E)** were determined after interference of LPA_2_-siRNA. Data were expressed as mean ± SD of three independent experiments. ^*^P < 0.05, compared with control. ^#^*P* < 0.05, compared with negative control.

LPA exerts various biological effects through different G protein-coupled receptors: LPA_1_–LPA_6_, and therefore we investigated which subtype of LPA receptors mediated MMP-9 expression. Since LPA_1_, LPA_2_, and LPA_6_ were highly expressed in THP-1-derived macrophages (Figure 5A), we separately knocked down the three LPA receptors using specific siRNAs and detected the MMP-9 expression with or without LPA treatment. As shown in Figure 5B, LPA_1_, LPA_2_, and LPA_6_ were effectively knocked down and LPA-induced MMP-9 expression and nucleus p65 levels were attenuated significantly by inhibition of LPA_2_ (Figures 5C–E). These data suggested that LPA_2_ was involved in LPA-induced upregulation of MMP-9 and activation of NF-κB.

## Discussion

In the present study, we found a positive correlation between LPA and MMP-9 concentrations in peripheral blood, which was in accordance with the overlapping distribution of ATX and MMP-9 in the human atherosclerotic plaques. Experiments *in vitro* clarified that LPA could effectively promote the expression and activity of MMP-9 mainly by binding to G protein-coupled LPA_2_ receptor that in turn activated NF-κB pathways.

Thinning of the fibrous cap is a crucial step that precedes plaque rupture and consequent secondary thrombotic events. A number of studies have revealed that SMCs contribute to cap thickening by producing elastin, collagen and other matrix components in stable atherosclerotic lesions; while during plaque destabilization, reduced matrix protein deposition resulted in weakening fibrous cap (Libby, 2013). In our study, patients with unstable plaques exhibit significantly elevated LPA/ATX levels in both circulation and vascular tissues, suggesting that LPA may contribute to atherosclerotic plaque instability. This is supported by recent reports demonstrating that LPA contributes to expansion of necrotic core by accumulation of ox-LDL induced macrophages (Schober and Siess, 2012), intraplaque hemorrhage by recruitment of mast cells (Bot et al., 2013), and thrombus formation by induction of platelet aggregation (Pamuklar et al., 2008; Bolen et al., 2011). Macrophages, mainly in necrotic cores (Gautier et al., 2009; Tabas, 2010), are an important source of MMPs for ECM degradation. We found a correlation in plasma and overlapped distribution in macrophage-rich lesions between LPA (ATX) and MMP-9, suggesting a relationship between LPA and MMP-9. Hence, whether LPA could participate in the regulation of the matrix composition through MMP-9 is of high interest. This study validated this hypothesis and established a detailed mechanism underlying LPA on MMP-9 expression in macrophages and provided a new perspective for the progression of atherosclerosis plaque destabilization.

MMP-9 (also known as gelatinase B, 92 kDa collagenase) is one of a family of endopeptidases that cleaves most ECM proteins, particularly collagen types IV and V (Pourmotabbed et al., 1994; Buraczynska et al., 2015). Strong evidence indicates that MMP-9 could contribute to wakening and rupture of atherosclerotic plaques. Consistent with our results, plasma MMP-9 in patients with UA, AMI, and carotid artery stenosis have been found elevated compared to controls (Kai et al., 1998; Ferroni et al., 2003; Renko et al., 2004; Tziakas et al., 2006; Taurino et al., 2007), even similar finding from urine (Fitzsimmons et al., 2007). High levels of MMP-9 are also found in plaque shoulders and regions of foam cells accumulation (Galis et al., 1994). Additionally, genetic studies have demonstrated associations between MMP-9 polymorphism and severity of coronary heart disease (CHD) (Pollanen et al., 2001; Blankenberg et al., 2003). The expression and activity of MMP-9 are regulated on several levels, including transcription, secretion, activation, and inhibition (Vandooren et al., 2013). In our study, Act.D and CHX experiments revealed that LPA-induced MMP-9 expression was up-regulated at the de novo synthesis of mRNA - transcriptional level. A variety of transcription factors for regulating MMP-9 are investigated, such as NF-κB (Dilly et al., 2013; Tseng et al., 2013; Yan et al., 2016), activator protein-1 (AP-1) (Lian et al., 2015; Yang et al., 2016), specificity protein 1 (Sp-1) (Murthy et al., 2012) and peroxisome proliferator-activated receptor-γ (PPARγ) (Lee et al., 2009; Chen et al., 2013). We showed that LPA-mediated MMP-9 expression in macrophages is dependent on NF-κB signaling through inhibitor experiments and promoter analysis.

As we know, six LPA receptor subtypes (LPA_1_–LPA_6_) have been discovered so far and mediate the diverse physiological effects of LPA. The expression profiles of LPARs are often different from different cell types. We displayed the distribution of LPA_1_–_6_ in THP-1-derived macrophages and found LPA_1_, LPA_2_, and LPA_6_ mRNA were highly expressed. Growing evidence has implicated that LPARs involved in pathophysiologic progression of atherosclerosis. LPA promotes smooth muscle progenitor cell recruitment for neointima formation and releases proinflammatory cytokines from ECs to heighten atherosclerotic plaque burden in an LPA_1_- and LPA_3_-receptor dependent manner (Subramanian et al., 2010; Zhou et al., 2011). Zheng et al. (2001) show that LPA stimulates T lymphocytes migration via LPA_2_. Sumida et al. (2010) indicate that Lpar4^−/−^ mice exhibit embryonic lethality in association with hemorrhage, suggesting that LPA_4_ may account for post-development blood vessel formation. LPA_5_ in particular has been reported to mediate LPA signals leading to platelet activation and aggregation (Williams et al., 2009). Almost every subtype of primary or cultured vascular cells exhibits some responses to LPA via LPARs (Smyth et al., 2014). In this study, we highlighted that LPA_2_ was responsible for LPA-induced NF-κB activation and MMP-9 expression in macrophages, which may provide promising target for therapeutic intervention of atherosclerosis.

Nevertheless, several limitations of the study are worth mentioning. Firstly, the sample size for cardiovascular events (*n* = 48) and coronary plaques (*n* = 3) was both small and correlation between LPA and MMP-9 plasma levels might be validated in a larger patient cohort. Secondly, the molecular mechanism by which LPA induced MMP-9 expression was not explored and further *in vivo* and appropriate animal models remained necessary. Thirdly, we detected LPA distribution in the plaques by ATX antibody staining due to the instability in LPA measurement caused by plaques acquisition and storage. Indeed, LPA levels from heterozygous ATX-null mice were approximately half those from wild-type mice (Tanaka et al., 2006).

In summary, this study demonstrated synchronous increases of LPA and MMP-9 in peripheral blood and overlapping distribution of them in local atherosclerotic plaques. The vitro experiments in macrophages revealed that LPA induced MMP-9 in a time- and dose-dependent manner, mainly by LPA_2_ to activate NF-κB signaling pathways. Therefore, intervention in LPARs may be an effective therapeutic strategy to target plaque destabilization.

## Author contributions

XC, XfC, FW, and CG conceived and designed the research; CG, HW, and ZZ performed the experiments; CG and FW analyzed the data. CG, XC, and XfC wrote and revised the manuscript.

### Conflict of interest statement

The authors declare that the research was conducted in the absence of any commercial or financial relationships that could be construed as a potential conflict of interest.
